# Motion and Trajectory Constraints Control Modeling for Flexible Surgical Robotic Systems

**DOI:** 10.3390/mi11040386

**Published:** 2020-04-07

**Authors:** Olatunji Mumini Omisore, Shipeng Han, Yousef Al-Handarish, Wenjing Du, Wenke Duan, Toluwanimi Oluwadara Akinyemi, Lei Wang

**Affiliations:** 1 Research Centre for Medical Robotics and MIS Devices, Shenzhen Institutes of Advanced Technology, Chinese Academy of Sciences, Shenzhen 518055, China; yousef@siat.ac.cn (Y.A.-H.); wj.du@siat.ac.cn (W.D.); wk.duan@siat.ac.cn (W.D.); tolu@siat.ac.cn (T.O.A.); 2 CAS Key Laboratory for Health Informatics, Shenzhen Institutes of Advanced Technology, Chinese Academy of Sciences, Shenzhen 518055, China; 3 Institute of Microelectronics, Chinese Academy of Sciences, Beijing 100007, China; hanshipeng@ime.ac.cn; 4 University of Chinese Academy of Sciences, Shenzhen 518055, China; 5 Shenzhen College of Advanced Technology, University of Chinese Academy of Sciences, Shenzhen 518055, China

**Keywords:** snake-like robots, inverse kinematics, minimally invasive surgery, motion control, robot dynamics, trajectory planning

## Abstract

Success of the da Vinci surgical robot in the last decade has motivated the development of flexible access robots to assist clinical experts during single-port interventions of core intrabody organs. Prototypes of flexible robots have been proposed to enhance surgical tasks, such as suturing, tumor resection, and radiosurgery in human abdominal areas; nonetheless, precise constraint control models are still needed for flexible pathway navigation. In this paper, the design of a flexible snake-like robot is presented, along with the constraints model that was proposed for kinematics and dynamics control, motion trajectory planning, and obstacle avoidance during motion. Simulation of the robot and implementation of the proposed control models were done in Matlab. Several points on different circular paths were used for evaluation, and the results obtained show the model had a mean kinematic error of 0.37 ± 0.36 mm with very fast kinematics and dynamics resolution times. Furthermore, the robot’s movement was geometrically and parametrically continuous for three different trajectory cases on a circular pathway. In addition, procedures for dynamic constraint and obstacle collision detection were also proposed and validated. In the latter, a collision-avoidance scheme was kept optimal by keeping a safe distance between the robot’s links and obstacles in the workspace. Analyses of the results showed the control system was optimal in determining the necessary joint angles to reach a given target point, and motion profiles with a smooth trajectory was guaranteed, while collision with obstacles were detected *a priori* and avoided in close to real-time. Furthermore, the complexity and computational effort of the algorithmic models were negligibly small. Thus, the model can be used to enhance the real-time control of flexible robotic systems.

## 1. Introduction

The da Vinci surgical robotic system, introduced two decades ago, has given rise to a rapid paradigm-shift in minimally invasive surgery (MIS). Recently, flexible prototypes have been developed by emulating bio-inspired creatures for robot-assisted diagnostic, surgical, and therapeutic procedures [1]. While robotic mechanisms with soft actuation and deformable joints are generally used in the context of flexible robotics, rigid serial-link robots have also been proposed and adapted to enhance MIS procedures that involve intrabody and intraluminal navigations [2]. While the robots are capable of flexible intrabody movements in certain human cavities, links in the prototypes that have redundant parts can be utilized for effective obstacle and singularity avoidance by using operative kinodynamics model [3,4]. Some existing surgical robotic systems, such as Zeus and da Vinci robotic platforms, adopt parallel-link design mechanisms for flexible access during surgical interventions. While navigating the parallel mechanisms requires passive control systems, recent advances in flexible MIS have shown that serial-link prototypes, such the da Vinci^®^ Sp (Intuitive Surgical, Sunnyvale, CA, USA) and Flex Robotic (Medrobotics Corp., Raynham, MA, USA) surgical systems, can perform MIS. Flexible prototypes are designed for a fast, safe, and precise stratagem needed for intrabody navigation during surgery interventions in confined anatomical areas of the human body. Some recent advancements regarding MIS and single-port orifical surgery were documented in Simaan et al. [4]. In addition, Simaan et al. [5] discussed MIS robots that were designed with unique features for the elimination of abdominal or rectal wall aggression and a reduction of postoperative trauma. However, a major issue is the lack of motion and force constraints, which hinder the effective control of the multisegmented robots during flexible access surgery.

Applications of flexible robotic systems with snake-like parts is still in its infancy for MIS. The existing prototypes utilize discrete or continuous joint mechanisms for navigation. Hence, safe and efficient clinical procedures with a flexible robot requires precise constraint models for motion and teleoperation control [6]. This involves the development of timely and accurate kinematics and dynamics models to enhance the precise point-to-point navigation of flexible robotic systems for intrabody manipulation [7]. In teleoperated surgery, the transmission of force feedback between the surgeon and surgical room requires the availability of reliable force data and protocol communication. Su et al. [8], proposed using a neural network for bilateral teleoperation with an enhanced robot tool identification and calibration. Constraints control modeling in flexible robotic mechanisms requires the intricate simultaneous analysis of the robot’s links and its workspace. The presence of multiple degrees of freedom (DoFs) in flexible robots enhances the dexterity of the robot’s links with snake-like navigation along flexible paths. However, this inordinateness leads to link or joint redundancy with the existence of more DoFs than what is needed to reach different manipulable areas in a low-dimensional space. Thus, developing a holistic motion control system is required for navigation using a flexible robotic mechanism in its operational work area. This involves designing timely and accurate kinematic and dynamic constraints with an effective path analysis for obstacle detection and avoidance during point-to-point navigation. Concerns are being raised regarding the acceptance and application of flexible robotic systems due to a lack of the appropriate and precise models required for the constraints control of flexible robots during MIS. Recently, Su et al. [9] developed an improved constraint control system with a human–robot collaborative model for the teleoperation of a redundant MIS robot. The control system includes an integrated compensator models for the enhancement of surgical precision at the tooltip, and to guarantee motion constraints. Similarly, several methods have been proposed, developed, and adapted for modeling the kinematics, dynamics, and teleoperation constraints control in flexible snake-like robots [1,4,5,6]. Nonetheless, research into coherent methods that can intelligibly combine the individual constraints control for the navigation of flexible surgical robots during intrabody operations still displays paltry progress [10]. To alleviate this, synergizing the kinematics and dynamics for modeling the required constraints control remains important open research. This is vital for the steady and consistent navigation of surgical robots along flexible and complex pathways.

Modeling kinematics and dynamic constraints without a loss of transparency and accuracy in master–slave surgical robots remains a challenging task and it has attracted research interests in recent years [6,11,12]. In general, kinematics resolutions basically involve defining apt relationships between the joint and Cartesian spaces of a robot. Forward kinematics (FK) involves modeling the coordinates of a robot’s links and end-effector based on a given joint vector, while the idea in inverse kinematics (IK) is to obtain the joint angles with which an end-effector of the robot can move along via-points of a trajectory. Since snake-like robots are kinematically redundant [6]; several methods have been proposed for achieving timely and accurate IK resolution [13]. Similarly, developing dynamic control models involves analyzing the disparities that occur between the motion and respective generalized forces at each joint of a robot. Lagrangian and Newton–Euler formulations are two conventional methods developed for dynamics modeling in serial-link robotics [14,15]. Variations of both methods have been reported in several works. Nonetheless, the high complexity and computation cost of each variant are common cons when used for high-DoF flexible robots [16,17]. Since joint forces are dependent on the physical limits of their actuators [18], the motion and force profiles of unique joints are investigated for point-to-point convergence of the robot during operation.

In this study, we designed a novel control system that could enhance both the motion and trajectory constraint control for the navigation of redundant snake-like robots along a flexible pathway during intraluminal surgery. To achieve this, a kinodynamics model was designed for solving the kinematics and dynamics of via-points established between the initial and target points on a surgical pathway, while a normalized cycloidal trajectory with boundary conditions was adapted for smooth and continuous point-to-option motion. Finally, the control system was enhanced with a switching method that decided alternative poses that were used to avoid collisions with obstacles. The main contributions of this study are:

(a) The computational modeling of motion constraints for snake-like robotic navigation during flexible surgical access. The model was based on a geometry-based inverse kinematics approach and it was also improved for dynamics resolution, trajectory planning, and obstacle avoidance.
(b) Development and implementation of the constraint models in motion and trajectory control software developed for the operation of flexible surgical robots.

The remainder of this paper is organized such that a brief review of studies on the design and control of flexible snake-like robots is presented in Section 2 and the structural design of a new prototype proposed for radiosurgical interventions is discussed in Section 3 along with the formulation of the kinodynamics and trajectory planning models proposed for the motion control of the flexible robot. The trajectory technique includes geometric modeling done for smooth and continuous navigation of the flexible robot and a collision avoidance scheme in cases of obstacle presence in the robot’s workspace. The simulation of the flexible robotic model and the implementation of the proposed control system are discussed along with the validation results from some case studies in Section 4. Finally, a conclusion of the study and directions for future work are briefed in Section 5.

## 2. Literature Review

Link flexibility and redundancy are vital features of continuum and snake-like robots, which makes them suitable in a wide range of surgical applications. A sizable number of flexible robotic prototypes have been designed following the introduction of the da Vinci robot and the recent advances in surgical interventions. In the earlier part of this millennium, a prototype for a highly dexterous snake-like robot was proposed for teleoperated surgery along the throat and upper airway, and it marked a vital point in adopting snake-like robots for flexible access surgery [19]. The integrated robotic platform is characterized by three-armed distally dexterous mechanisms that are designed for the manipulation of surgical devices in a confined space. The motivations for the surgical application of flexible robotic systems in humans and clinical trials lead to the commercialization of the Viacath snake-like robotic system (EndoVia Medical Inc., Norwood, MA, USA). The flexible surgical tool consists of a control platform that surgeons use to remotely manipulate the surgical instruments deep inside a patient’s body. After the acquisition of the system by Hansen Medical (Mountain View, CA, USA) in 2005, the Viacath system became one of the few dexterous surgical robots commercialized for single-port intraluminal interventions [20,21]. Furthermore, the application of a flexible robotic platform, described as a highly articulated flexible robot, was proposed for less invasive cardiac surgery. The robot has astounding features that are uncommonly found in conventional MIS robots [22]. As a proof of concept, models of the snake-like robot were developed for flexible percutaneous intra-pericardial interventions in Ota et al. [23] and Thakkar et al. [24]. Recently, Medrobotics Corp. (Raynham, MA, USA) developed a snake-like robotic system with flexible navigational access for hard-to-reach tissues in the mouth, throat, rectum, and colon during gynecological and thoracic procedures [25].

Teleoperation is a common technique that is being adopted for the manipulation of flexible surgical robots during MIS procedures. The teleoperation control standard requires effective underlying models for the fast, safe, and accurate navigation of a robot’s multiple links during flexible access surgery. Currently, the follow-the-leader approach has been proposed for motion modeling and control of flexible robotic systems, especially ones based on concentric tube and snake-like mechanisms [26]. The approach requires resolving kinematics of links in the robot’s lead module and transmitting the IK solution directly to subsequent modules for navigation via flexible pathways. Several IK models have been proposed for IK resolution in redundant robots [13]. These can be categorized into analytical-based solutions wherein algebraic methods are used to solve closed-form and geometric systems of equations, and numerical-based solutions that involve iteratively minimizing kinematics errors. A more detailed review has been presented in Seah et al. [6]. In general, algebraic methods involve the expansion of complex trigonometric equations due to high the transcendence in joint variables and symbolic expressions, while the existence of singular points in a robot’s workspace limits the latter. Precise motion control in multi-articulated robots requires consistent coordination of the different components driving the robotic links, camera(s) used for visualization, and surgical instruments. Dynamics control includes modeling timely and efficient methods for motion control, planning along a given trajectory, and computational analysis of the generalized forces acting on a flexible robotic system during surgery. In serial-link robots, Newton–Euler and Lagrangian formulations remain the basic techniques used for dynamics modeling [16,27], and several methods have been proposed based on them. For instance, in Saha [15], Bernoulli–Euler beam theory was utilized to analyze the effects of generalized forces in modeling the dynamic motion and control of a flexible robot. Newton–Euler formulations are recursive methods used for modeling generalized forces acting on a robot. This involves analysis of the robot’s motion profile with respect to coordinates of frames at the fixed-base and free-moving ends of the robot. The recursive Newton–Euler system of equation proposed in Luh et al. [14] was adapted in the efficient constraint models described for continuum robots in Ali [28].

Flexible-joint robots are now commonly used in different fields, including healthcare services. For this, Dong et al. [29] developed a force-free control model for a flexible-joint robot, and experiments were conducted to verify friction and gravitational force-free situations during human–robot interactions. Another study in a closer domain is the improved human–robot collaborative control scheme proposed for teleoperated MIS in Su et al. [8]. However, this study either did not investigate the motion and force profiles during point-to-point navigation or did in domains that were different from flexible robotic surgery. Nonetheless, the underlying techniques, for instance in References [14,30], can be adapted with respect to a robot’s structure, required navigation, and desired trajectory with a degree of dynamic stability during surgical procedures. In a recent study, efficient dynamic constraint modeling was presented for the effective control and teleoperation of flexible snake-like robots in Omisore et al. [30]. This model includes mapping the workspace of master and slave devices based on their kinematics resolution and adaptation of a higher other-motion profile for a direct analysis of the generalized forces acting on the robot’s joints. However, the IK approach can suffer from the singularity problem, and thus, the stability of the joint torques is not guaranteed. Furthermore, only the first two derivatives of the position data are utilized for the dynamic model. Generally, most studies on continuum and snake-like surgical robots do not give due attention to analyzing the motion/force interactions during flexible access surgery.

Besides kinematics and dynamics modeling, path and trajectory analyses are still open research points regarding the use of snake-like robots for flexible access surgery. This is said to be because link–obstacle interactions make it hard to generate an optimal trajectory in surgical areas cluttered with abdominal organs. Trajectory planning includes modeling smooth and shortest routes between two points in the robot’s workspace. In the last two decades, several algorithms have been proposed for trajectory analysis in robotics. Lozano-Perez [31] proposed a primitive method of path planning using an object’s pose as single points in the robot’s workspace. If objects are deemed as via-points of an end-effector, its consecutive pose information is sufficient for motion and trajectory planning. Angeles et al. [32] presented a trajectory planning method for motion along a continuous path in a robot’s configuration space. This requires specifying pose values of the robot’s end-effector using via-points as targets in the workspace. In Parsa et al. [33], an interpolating polynomial was used for the trajectory planning of a three-DoF manipulator such that smooth and jerk-free motion is steadily produced. Similarly, Dasgupta et al. [34] proposed a variational approach for path planning in hyper-redundant manipulators. Recently, Kuntz et al. [35] presented a sampling-based motion planner for flexible surgical robots based on its manipulations outside the body. Typically, most mechanisms consist of serially-connected modules that are capable of bending in unique planes. Liu et al. [36] designed an MIS pediatric surgical robot, with the latter having nine-DoF flexible robotic arms for dexterous interventions in a confined and narrow workspace. Another important aspect of the flexible robots designed for MIS is collision avoidance. Thus, Sun et al. [37] presented a safe motion planning method for an imprecise flexible robot by minimizing the collision probability. Similarly, EMG-based analytics is being adapted for robotics motion control and related teleoperation procedures, such as tool identification and calibration [38,39,40].

Similarly, artificial intelligence has been applied to solve motion constraints and trajectory planning problems in flexible robots. Agarwal [8] applied fuzzy C-means, an AI-based approach, for planning the trajectory of a four-DOF redundant manipulator. The clustering model was based on weighted within-scatter and between-cluster metrics for the robot, with a manipulability index taken as the performance criteria. Aside from fuzzy methods, models based on a neural network (NN) and a genetic algorithm were utilized for the trajectory control and analysis of flexible robots. The network provides fast methods for trajectory and path control. Chen et al. [41] designed a 3D neural model for a safety-enhanced trajectory in a workspace with the consideration of a minimum sweeping area. A memory neural network was used in Berni et al. [42] for the path optimization of a three-DoF robotic arm. Furthermore, artificial neural network approaches [6,43] are used in trajectory planning. For instance, a method based on Hermite was recently proposed for the motion planning and obstacle avoidance of serially redundant robots. Models based on genetic algorithm have been applied for generating a cubic interpolation polynomial, such as that given in Chen et al. [44]. The history of the task space trajectory was approximated and the interpolation points were obtained using the proposed genetic algorithm. Schmitz et al. [4] combined a genetic algorithm with a recorded suturing and anatomical data to control the four distal DoFs of a flexible robot over a predefined trajectory. Modeling obstacle avoidance for flexible robots with multiple DoFs is challenging; yet, a deep recurrent NN-based model was developed and investigated for a redundant serial-link robot in Xu et al. [45]. The recurrent network established the effective avoidance of static and dynamic obstacles. Recently, a vision-based obstacle avoidance approach was developed based on a sequential NN [46]. The study was focused on an aerospace application but similar models can be adopted for flexible robotic surgery. Furthermore, a neural-learning-based control model was proposed for constraints control robotic systems with flexible joints in He et al. [47]. A drawback of intelligent-based control systems is a requirement of training that hinders behavioral cloning. Since the control system is mainly used to navigate a robot under a planned motion sequence, such control systems might be impractical when used in a new setting. Furthermore, network-based techniques usually require objective methods for defining an optimal network topology. Thus, while designing and training a network takes time, learning requires a high amount of storage and control errors are unpredictable.

There is a great need to focus efforts on model-based motion and trajectory control models for the safe, precise, and collision-free navigation of snake-like robots. Harmonic potential functions were used to compute collision-free paths in redundant robots [48,49]; however, the approach may likely cause manipulators to be trapped in local minima. Parsa et al. [34] extended the use of an interpolating polynomial to generate a smooth path between initial and final points such that robot–obstacle collisions were avoided. A general formulation of a trajectory planner includes collision avoidance as a sub-problem. Thus, a better method is to plan the trajectory with index measures taken to maximize the robot’s manipulable area. A pragmatic observation regarding the reviewed studies shows that the intrabody navigation of snake-like robots requires constraints modeling for efficient kinematic and dynamic control, optimal trajectory planning, redundancy resolution, and singularity and collision avoidance for safe intrabody navigation [4]. While most authors usually focus on modeling each of those constraints individually, some groups of robotics researchers have developed control systems that include both kinematic and dynamic models. Similarly, stipulations for optimal trajectory analysis and collision avoidance have also been considered but there are only a few examples of this. Therefore, the development of motion control systems with modular interfaces for inclusiveness and the concurrent operability of each constraint model is still lacking across all existing studies.

## 3. Robotic Design and Motion Control System

### 3.1. Design of the Flexible Snake-Like Robot

**Figure 1:**
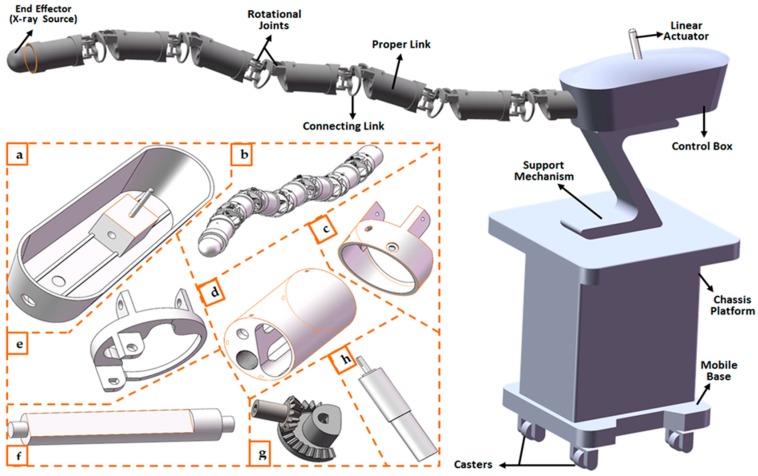
Design of the flexible snake-like robot: (**a**) translational joint mechanism, (**b**) flexible links with rotational joints, (**c**) end caps, (**d**) proper link, (**e**) connecting link, (**f**) inter-module and intra-module link connectors, (**g**) rotational joint mechanism, and (**h**) linear actuator.

The snake-like robotic design, presented in Figure 1, is part of a developmental study proposed for the intrabody delivery of radiation doses during MIS radiosurgical gastrointestinal intervention. The flexible modular mechanism uses consecutive links and orthogonally paired joints for point-to-point navigation of its end-effector along a given pathway. Each module has a pair of links connected by a rotational actuator, which is connected to the subsequent module by another rotational actuator. That is, paired-links within each module are a set of proper and connecting links. Proper links are characterized by having a long length and two end-caps fixed at both ends for connection with the shorter connecting links. Each rotational joint is driven by an actuator with a brushless DC micro made-up of a rotor and stator. The flexible robotic mechanism is directly connected to a mobile base built with a chassis platform and casters. Interventional tasks involve steering the flexible mechanism with serpentine poses that can be fitted for intrabody navigations. In this study, the control procedure was built by taking a unique $P^{n}R$ configuration of the robotic model. This includes the prismatic joint, which controls the translational motion of the robot’s end-effector, and separate analyses were carried out to solve the motion constraints of the n-DoF revolute joints. The remaining parts of this section are focused on modeling an all-inclusive control system for the motion and trajectory control of a typical $P^{n}R$ snake-like robotic model. For this purpose, kinematic and dynamic constraints models were first developed for point-to-point motion control of the flexible robot, then a trajectory model was developed for smooth navigation and that avoids collisions with obstacles detected in the robot’s workspace.

### 3.2. Kinodynamics Modeling

**Figure 2:**
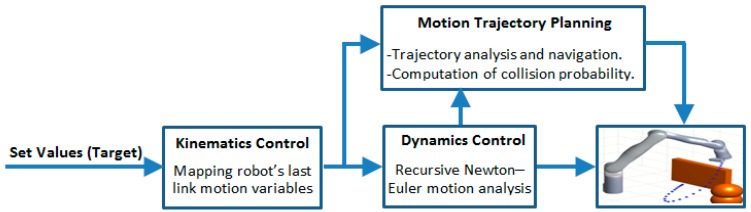
Operational flow of the control systems proposed for the flexible snake-like robot.

Following the operational flow of the control systems presented in Figure 2, modeling the kinodynamics of the flexible snake-like robot was approached by designing fast and accurate kinematic and dynamic models for solving the motion constraints of the robot. Kinodynamic models are typically based on models derived for the constraints control of the robot, as explained below.

#### 3.2.1. Kinematics Constraints Resolution

Kinematic constraints resolution involves the derivation of a fast and accurate model for transforming Cartesian space to joint space, which is necessary for motion control in robotics. Kinematics resolution is vital for the point-by-point motion of a flexible surgical robot, and FK has been widely solved using direct frame translation and Denavit–Hartenberg (DH) conventions. Thus, our focus was to derive the IK modeling in this study while the DH matrix in Equation (1) adopted FK, where $T_{e}\left(\theta \right)$ is the cumulative product of the transformation matrices from the base joint of the robot to the *n*th joint over a given set of joint variables: $\theta =\left[\theta_{1},\;\theta_{2},\;\ldots ,\;\theta_{n}\;\right]$:

(1) $$T\left(\theta \right)=\overset{n}{\underset{i=1}{\prod }}\left(\begin{array}{cc} \begin{array}{cc} cos\theta_{i} & -sin\theta_{i}cos\alpha_{i}\\ sin\theta_{i} & cos\theta_{i}cos\alpha_{i} \end{array} & \begin{array}{cc} sin\theta_{i}sin\alpha_{i} & a_{i}cos\theta_{i}\\ -cos\theta_{i}sin\alpha_{i} & a_{i}sin\theta_{i} \end{array}\\ \begin{array}{cc} 0\; & sin\alpha_{i}\\ 0\; & 0\; \end{array} & \begin{array}{cc} cos\alpha_{i} & \;d_{i}\\ 0\; & \;1\; \end{array} \end{array}\right).$$

The first link slides in and out horizontally along the z-axis of the flexible arm’s base frame, while the remaining links are revolute joints. The second link has an orthogonal joint, which has a frame rotation that is perpendicular to both the base frame and the subsequent links. Finally, all other links are connected using an orthogonal twisted joint starting from the first proper joint. Following the geometric method in Su et al. [9], the last n−2 DoFs are made to rotate in a single rotation axis. The IK method for obtaining the robot’s joint vector for given target points are derived as follows:

(a) Determination of the Joints’ Positions

Given a target position $P_{T}{}^{{ℜ}^{3x1}}$ as a point in 3D coordinates, we assumed a fixed value for the prismatic joint since it does not affect the serpentine movements of the snake-like robot. Then, the angle at the first revolute joint (φ) is computed with Equation (2). This puts the second revolute joint in a diagonal direction relative to the target point ($P_{T}$), where {a,b,c} are vertices of a triangle formed by coordinates of the first and second revolute joints, and the reflection of $P_{T}$ on the XY-axis respectively; A is area of $∆b\hat{a}c$.

(2) $$\varphi =\pm \;\left[2*atan2\left(4A,\;{\left(b+c\right)}^{2}-a^{2}\right)\right]$$

Knowing φ, the coordinates of second revolute joint $\left(P_{R2}\right)$ can be computed as the ratio of a vector ($V_{x,y,z}$) that reaches $P_{T}$ as in Equation (3), where: $V_{x,y}={\left[P_{T}\left(x,y\right)+P_{R1}\left(x,y\right)\right]}^{T}$ and $L_{2}$ is the length of the second link. Furthermore, $sgn\left(P_{R}\left(x\right)\right)$ is always positive, but $sgn\left(P_{R2}\left(y\right)\right)=sgn\left(P_{TP}\left(y\right)\right)$. The minimum assumption for the existence of a solution is that the distance between $P_{R2}$ and $P_{T}\leq \sum l_{i}$, $s.t.\sum l_{i\;}$= sum of length of the remaining links. To calculate the positions of other joints, $P_{R2}$ and $P_{T}$ are transformed to a 2D space using Equation (4), where $P_{Ri}$ is the coordinate of *i*th intermediate joint in 2D, and ε is the error loss derived in Omisore et al. [13]:

(3) $$P_{R2}={\left[\begin{array}{cc} \frac{L_{2}}{\sum_{j=1}^{m}L_{j}}*abs\left(V_{x,y}\right) & d_{1} \end{array}\right]}^{T},$$

(4) $$P_{Ri}^{{ℜ}^{2x1}}={\left[\begin{array}{cc} \sqrt{P_{Ri}{\left(x\right)}^{2}+P_{Ri}{\left(y\right)}^{2}} & P_{Ri}\left(z\right) \end{array}\right]}^{T}+\varepsilon .$$

**Figure 3:**
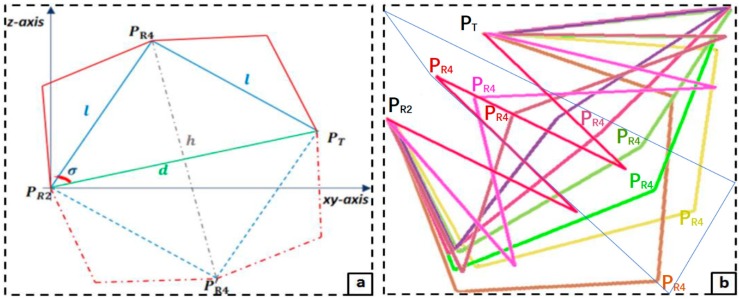
Analysis of the obstacle avoidance in the inverse kinematics (IK) model: (**a**) locating the mid-point for the IK computation and (**b**) alternative poses of a flexible four-DoF robot based on different values of the base angle (σ).

Navigation of the remaining four links can be viewed in a planar XY/Z axis. These links can be sub-divided as two equidistant two-link mechanisms whose common joint is a mid-point selected arbitrarily along the ash line with height h, as typified with blue lines in Figure 3a. By varying the coordinates of the mid-point ($P_{R4}$), the locations of the virtual points $P_{R3}$ and $P_{R4}$ change with the base angle; thereby, the two two-link mechanisms $\left(P_{R2},\;P_{R3},P_{R4}\right)$ and $\left(P_{R4},P_{R5},\;P_{T}\right)$ can attain diverse poses, as shown Figure 3b. Vertices locations for any of the poses can be obtained with the triangulation expressions in Equations (5) and (6). The base angle (σ) is computed such that each mechanism forms an isosceles triangle, while d and l are the length of either of the isosceles sides of the triangle and the length of the base side, respectively.

(5) $$P_{Ri}^{{ℜ}^{2x1}}=P_{R2}+\left(l*\left[\begin{array}{c} cos\left(\sigma \right)\\ sin\left(\sigma \right) \end{array}\right]\right)$$

(6) $$\sigma =cos^{-1}\left(\frac{\;d^{2}}{2*d*l}\right)$$

(7) $$\theta_{i}=atan2\left(P_{Ri}\;⊗\;P_{Ri}^{\prime },\;\;P_{Ri}\;ʘ\;P_{Ri}^{\prime }\right)$$

(b) Computation of Joint Angles

Since the prismatic offset (∅) depends on the prismatic joint, and the equation for φ was already given in Equation (2), the remaining joint angles $\left\{\theta_{1},\ldots ,\;\theta_{4}\right\}$ are computed as angular displacements between consecutive locations of joint pairs in the XY/Z axis. Suppose the coordinates of the tip of the *i*th link is $P_{Ri}^{\prime }$ when $\theta_{i\geq 1}=0$ such that the needed angle $\theta_{i}$ that ensures the tip is at $P_{Ri}$ is given as Equation (6), where {a⊗b, a ʘ b} are cross and dot products between the vectors, respectively. $PV_{Ri}^{\prime }$ is derived from the FK of the previous *i*−1 joint angles, and setting a value of $\theta_{i}$ = 0. Further, $PV_{Ri}^{\prime }$ and $PV_{Ri}$ are normalized with respect to a common origin $PV_{Ri-1}$. Finally, the joint angles $\{\emptyset ,\;\varphi ,\;\theta_{1},$ $\theta_{2},\;\theta_{3},\;\;\theta_{4}\}$ is an inverse kinematics solution that is needed to drive the flexible robot to the given target point $P_{T}$ in the robot’s workspace.

#### 3.2.2. Dynamics Resolution

The motion or path-tracking ability of the robot can be designated as a measure of how optimal motion and generalized forces are ensured in all parts (links and joints) of the robot. A compact model with partial derivatives of the non-linear motion variables was built to analyze the dynamic constraints of the snake-like robot during flexible path navigation. The recursive model is a new variant of the Newton–Euler’s approach in Luh et al. [11]. Considering the translational and rotational joint mechanisms of the snake-like model in Figure 1, the generalized forces acting on the link can be approximated using the state-space dynamics given in Equation (8), where $\tau_{e}\in {ℜ}^{6\times 1}$ is the actuator torque; $M\left(\theta \right)\in {ℜ}^{6\times 4}$ is a symmetric and positive definite matrix for the robot’s inertia; $C\left(\theta ,\dot{\theta }\right)\in {ℜ}^{6\times 1}$ is a vector containing centrifugal and coriolis terms; $G\left(\theta \right)\in {ℜ}^{6\times 1}$ is a vector for the moment due to gravity; and $\dot{\theta }$ and $\ddot{\theta }$ are higher motion variables, i.e., velocity and acceleration, respectively, for the joint:

(8) $$\tau_{e}=M\left(\theta \right)\ddot{\theta }+C\left(\theta ,\dot{\theta }\right)+G\left(\theta \right).$$

**Figure 4:**
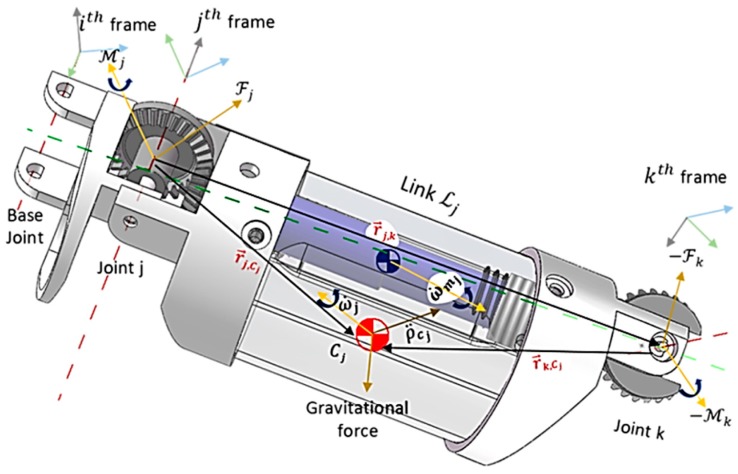
Newton–Euler Formulation for an arbitrary link in *ijk* coordinates.

The state-space system in Equation (8) can be used for both forward and backward recursions to obtain the generalized forces at the robot’s joints. In the former, the higher motion variables are computed up to acceleration at the last link. This involves maneuvering the frames attached to each joint, i.e., from the base link up to the last link in the robot. On the other hand, generalized forces at each joint are modeled using the backward recursion. Thus, direct evaluations of the state-space system in Equation (8) are done in forward and backward recursions. Considering $L_{j}$ in Figure 4 as an arbitrary link having its center of mass at $C_{j}$, if ${\vec{u}}_{j}$ is a vector at joint j to joint k(k∶=j+1) and aligns with $L_{j}$ in magnitude and direction, ${\vec{u}}_{j}$ can be described with respect to frame i at the robot’s base (i∶=j−1). Thus, the forward kinematic model is obtained with the generalized DH matrix by taking $a_{j},\;\theta_{j},\;\alpha_{j},$ and $d_{j}$ as parameters of link $L_{j}$ in Equation (1). Since the coordinate systems of frames attached to the robot’s links are orthonormal, any arbitrary point ${\vec{r}}_{j}$ along vector ${\vec{u}}_{j}$ in frame k can be defined with respect to frame j using the relation (r→ jj,k), as given in Equation (9), where R ijT  and P ij are obtained using Equations (10) and (11), respectively:

(9) r→ ij,k=R ijT(P ij)=[ajdjsin(αj) djcos(αj)],

(10) R ij=[cos(θj)−sin(θj)cos(αj) sin(θj)sin(αj)sin(θj) cos(θj)cos(αj)−cos(θj)sin(αj)0sin(αj)cos(αj)],

(11) P ij=[ajcos(αj)ajsin(αj) dj)].

During navigation, the motion of any link in the snake-like robot is assumed to completely depend on the kinematic resolutions of all subsequent links from the fixed base. Thereby, given that θ ji=1,…,n is the IK solution obtained for link $L_{j}$ along a given path, the velocities and accelerations of the link can be defined as time derivatives of the joints’ positions along the trajectory. Thus, the Newton–Euler equations can be used to determine the generalized forces acting on each link in the forward and backward recursions. Models of both forward and backward recursions are derived as presented in the following subsections.

(a) Forward Recursion

Since the initial velocity and acceleration of the base link are assumed, the higher motion variables at the robot’s joints are propagated outward from the base link up to the last link while navigating the robot. During the motion, the values of the variables are computed using Equations (12)–(16):

(12) ω jj={R ijT∗ω ii  J1   R ijT∗(ω ii+θ˙jz^o) Ji=2,…,n,

(13) ω˙ jj={R ijT∗ω˙ ii  J1   R ijT∗(ω˙ ii+θ¨jzo+θ˙jω ii×z^o) Ji=2,…,n,

(14) ρ¨ jj={R ijT∗(ρ¨ ii+ɗ¨j)+2ɗ˙jω ii×R ijTz^o+ω˙ ii×r→ ji,j+ω ii×(ω ii×r→ ji,j) J1R ijTρ¨ ii+ω˙ jj×r→ ji,j+ω ii×(ω ii×r→ ji,j) Ji=2,…,n,

(15) ρ¨ jCj=ρ¨ jj+ω˙ jj×r→ jj,Cj+ω ii×(ω ii×r→ jj,Cj),

(16) ω˙ imj=ω˙ ii+ƙr,jθ¨jz^ imj+ƙr,jθ˙jω˙ ii×z^ imj,

where $\dot{ɗ}$, ${\ddot{ɗ}}_{j}$, $\omega_{j}$, ${\dot{\omega }}_{j}$, and ${\ddot{\rho }}_{j}$ are the linear velocity, linear acceleration, angular velocity, angular acceleration, and linear acceleration of a *j*th frame in the robot’s joints; ${\ddot{\rho }}_{c_{j}}$ is the linear acceleration of the center of mass of link *j*; ${\dot{\omega }}_{m_{j}}$ is the angular acceleration of a rotor in the motor at the *j*th joint; ${ƙ}_{r,j}$ is the gear reduction ratio of the motor; ${\vec{r}}_{i,j}$ is a vector from the origin of frame *i* to frame *j*; ${\vec{r}}_{j,C_{j}}$ is a vector from the origin of frame *j* to the center of mass $C_{j}$; ${\hat{z}}_{o}∶={\left[\begin{array}{ccc} 0 & 0 & 1 \end{array}\right]}^{T}$ is a constant vector; and ${\hat{z}}_{m_{j}}$ is a unit vector used to describe the rotation axes of the rotor in *j*th motor with respect to an *i*th frame.

(b) Backward Recursion

Conversely, a given terminal force and moment, which is expected to act on the last link of the robot, are propagated inward from the last link down to the base link to compute the forces and moments acting on the initial links. In this step, results obtained for each link during the forward recursions are applied to determine the force ($F_{j}$) and moment ($M_{j}$) given in Equations (17) and (18), respectively, during backward recursions, where $m_{j}$ and ${\bar{Ɨ}}_{j}$ are the mass and moments of inertia taken at $C_{j}$, respectively, and are aligned with the output coordinate system of link *j*; ${Ɨ}_{m_{k}}$ is the moment of inertia of the rotor in the motor at the *j*th joint; and $\tau_{j}$ is the torque acting on the *j*th joint. In the final equation of the dynamic constraints model, the resultant generalized forces, otherwise taken as torque acting at the initial joints, are computed using Equation (19). For faster dynamics analysis during control navigation of the flexible robot, only one frame transformation is utilized for forward and backward recursions, unlike in Luh et al. [11]. However, frictional forces acting at the robot’s joints are not considered in this study.

(17) F jj=mjρ¨ jCj+R jkF kk

(18) M jj=2(ω jj×(Ɨ¯ jjω jj))−F jj×(r→ ji,j+r→ jj,Cj)+R jkM kk+ƙr,kθ¨kƗmkz^ jmk+Ɨ¯ jjω˙ jj+ƙr,kθ˙kƗmkω jj×z^ jmk

(19) τj={F jjR ijTz^o+ƙr,jƗmjω˙ imjTz^ imj  J1   M jjTR ijTz^o+ƙr,jƗmjω˙ imjTz^ imj Ji=2,…,n

### 3.3. Trajectory Planning and Obstacle Avoidance

The motion planning in flexible robotic systems is concerned with modeling an effective trajectory model that can steer the robot’s links from an initial point to a desired position in the robot’s operational workspace. Usually, motion planning involves finding an optimal trajectory in which certain via-points are generated for point-to-point navigation of the robot under a pre-defined motion profile. Since the links’ trajectories cannot be easily predicted in advance and may cause collisions with the obstacles present in the workspace, obstacles avoidance is another crucial area of motion planning and constraints control in flexible robotic systems. To this end, the kinodynamic constraints models were further refined to ensure a continuously smooth point-to-point navigation. Kinematic redundancy in such robots aids motion flexibility; thus, we take it as a ploy to detect and avoid collision with obstacles in the operational space during flexible navigational access [24].

#### 3.3.1. Motion Trajectory Planning

In flexible robotics, trajectory planning manages the computation of an interpolating sequence of desired via-points through which a robot’s end-effector can only be restricted to certain regions of interest [42]. Both the case of path analysis and navigation from an initial point through a sequence of points to a final point are vital for flexible route access during intrabody robotic surgery. Thus, the first task in motion trajectory planning is to define an optimal path, i.e., the shortest obstacle-free route between initial and final points, a robot will navigate. In path analysis, the shortest route between two points in a workspace is a straight line joining the points with a vertical, horizontal, or diagonal movement. Hence, given two points $\left\{P_{i},P_{j}\right\}$ as initial and target points in the manipulable area of a robot, its default path navigation can be parametrically given as Equation (20) if $p\langle \delta x,\delta y,\delta z\rangle =\vec{P_{i}P_{j}}$, where $\left\{P_{i},\;P_{j},\;\vec{P_{i}P_{j}}\right\}\in {ℜ}^{nx1}$:

(20) $$Path_{m}=P_{i}\langle x,y,z\rangle +p\langle \delta x,\delta y,\delta z\rangle .$$

Since the movement with p〈δx,δy,δz〉 is taken to produce a typical point-to-point motion, it is only considered optimal in an obstacle-free workspace. Thus, this path navigation strategy utilizes a set of the robot’s poses to move from a point $P_{i}$ to point $P_{j}$ in a free workspace. The poses are determined in the configuration space with respect to the robot’s geometric constraints. For efficient obstacle avoidance, the constraints control method could get the robot stuck, or abruptly change the motion profile of some links in the mechanism. This can damage a robot’s actuators. To avoid both scenarios, a multi-segmented path can be generated for trajectory planning. This will help to avoid jerks during the motion. In this study, a cycloidal trajectory is applied for steady and continuous motion. Analytically, if the flexible robot has to produce a motion by navigating through *n* via-points in T (s), the normalized profile of the motion’s trajectory, viz. the position and the first two higher orders of the robot’s motion, can be derived using Equations (21)–(23):

(21) $$q_{N}\left(t\right)=\left(\frac{t_{i}-t_{0}}{T}-\left(\frac{1}{2\pi }*sin2\pi \tau \right)\right)\vec{P_{i}P_{j}},$$

(22) $${\dot{q}}_{N}\left(t\right)=\left(1-cos2\pi \tau \right)\vec{P_{i}P_{j}},$$

(23) $${\ddot{q}}_{N}\left(t\right)=\left(2\pi *\;sin2\pi \tau \right)\vec{P_{i}P_{j}}.$$

The time taken to navigate the segment is obtained by scaling τ by a factor λ=T and translating with respect to the initial motion time. Thus, displacement of the *j*th joint for the *i*th segment can be defined as Equation (24), where $t_{i}$ is the time taken to navigate the *i*th segment:

(24) $$\theta_{j}\left(t\right)=\theta_{j}^{i}+\left(\left(\theta_{j}^{f}-\theta_{j}^{i}\right)*\|q_{N}\left(t\right)\|\right).$$

#### 3.3.2. Obstacle Detection and Avoidance

**Figure 5:**
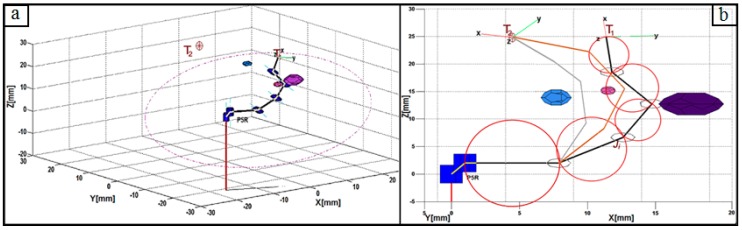
Analysis of the IK solution for obstacle (**a**) detection and (**b**) avoidance during motion planning.

A more complex scenario is path tracking in the presence of an obstacle(s). This scenario could be one where the obstacles intersect the straight-line or limits the freedom of one or more of the robot’s links. Modeling the path as a straight line becomes infeasible in the first case; instead, the path is modified to thread the convex hull [50] of the obstacle. This is not a typical problem in articulated arm robots unlike the latter case where obstacles could limit the operation of some links, thereby deflecting the robot’s path threading. As shown in Figure 5, the presence of obstacles in the workspace requires further analysis to obtain possible motions for each link in the snake-like robot. For this purpose, the IK model in Section 2 was enhanced with strategies for obstacle collision detection and avoidance.

(a) Collision Detection

Optimal mid and virtual points are needed to achieve a stable and collision-free point-to-point motion. Collision is analyzed in Figure 5a using a unique rotation axis of the robot’s links in the workspace. The internal organs in the human body are taken as obstacles during MIS procedures. In this study, polyhedral objects are generated in the robot’s workspace, as shown in the 3D view. Since the robot’s last four links have a unique operational plane whose orientation depends on the respective offset ∅ and angle φ of each of the first two links, during each stepwise movement, only a polygonal area of the polyhedral obstacles that falls onto the operational plane is required for collision detection. While the via-points of a desired trajectory are sequentially set as input for motion planning and control, the probability of collision *(*$PoC_{l,k}$) between *l*th link of the robot’s with any *k*th obstacle in the workspace is determined using Equation (25). In the analytical method, it is assumed that each link and each obstacle in a manipulable area of the robot’s workspace are circumscribed in unique circles along a rotation axis of the *l*th link, where $∆:k_{X}-l_{X}^{i}$, and $\left[r_{l},\;r_{o}\right]$ are independent radiuses of the links and obstacles in the workspace, respectively.

(25) $$PoC_{i,k}=\{\begin{array}{c} \begin{array}{cc} if\;\left(\left(\sqrt{{\left({∆}_{x}\right)}^{2}+{\left({∆}_{y}\right)}^{2}+{\left({∆}_{z}\right)}^{2}}\right)-r_{l}^{i}\right)\leq \;r_{o} & 1 \end{array}\\ \;\;\;\;\;\;\;\;\;\;\;\;\;\;\;\;\;\;\;\;\;\;\;\;\;\;\;\;\begin{array}{cc} else & \;\;\;\;\;\;\;\;\;\;\;\;\;\;\;\;\;\;\;\;\;\;\;\;\;\;\;\;\;\;\;\;\;\;\;\;\;\;\;\;\;\;\;\;\;\;\;0 \end{array} \end{array}$$

(b) Collision Avoidance

A collision-free path in a plane means that all parts of each robot’s links are at a safe distance away from all polygonal obstacles in the plane. Thus, for the robot’s links to move from $T_{1}$ to $T_{2}$ in the presence of obstacles in an operational plane, all possible values for the midpoint $P_{R4}$ and the virtual points required to reach $T_{2}$ are determined using the IK model. For $PoC_{l,k}>0$, the position of $P_{R4}$ along *h* is modified to ensure all parts of the links will not be in contact with the polygonal area of the obstacles for a point-to-point motion. Collision probabilities ($PoC_{l,k}$) for all sets of the mid-point and corresponding virtual points that could move the robot’s effector across the via-points are drawn into an array. Each $PoC_{l,k}$ is a total value defined over all the links (n-DoF) and obstacles, where this is done for all poses (IK solutions) with which the flexible robot can achieve a movement $P_{i}\to$ $P_{j}$. Thus, all are $PoC_{l,k}$ ranked, the optimal tuple is selected as the one with the lowest $PoC_{l,k}$ value, and the associated IK solution is utilized for the $P_{i}\to$ $P_{j}$ movement in the operational plane. Examples of this case are the ash and red poses in Figure 5b. In cases with more than one obstacle, all $PoC_{l,k}$ values could be greater than 0 where collision is just from a single link. Thus, the angle of the first revolute joint (φ) is re-computed to change the operational plane, viz. the black pose in the figure.

## 4. Implementation and Validation Results

**Figure 6:**
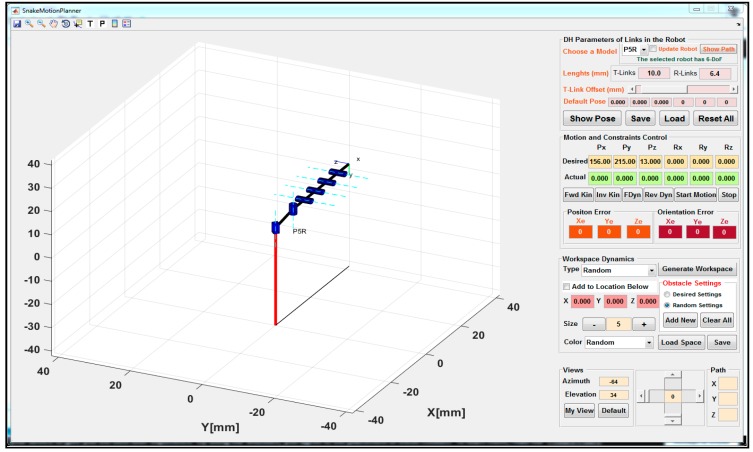
Default view of the motion control system.

In this study, a typical $P^{n}R$ (n = 5) prototype of the snake-like robotic model in Figure 1 was simulated with the Robotics Toolbox [51] in Matlab (MathWorks, Inc., Natick, MA, USA). The flexible robot had six inter-connected links and was capable of spatially flexible and dexterous motion. The robotic model’s DH parameters were chosen such that the first link (connected via prismatic joint) had a length of 100 mm and a maximum joint offset (ɗ) of 2 mm, while the remaining five links were connected via revolute joints. Each of the latter was made up of a proper link (53 mm in length) and an interconnecting link (11 mm in length) connected with a dummy joint; thus, a unique length of 64 mm in total. While only the first two links connected with a revolute joint had an orthogonal joint (thus, their rotation axes were perpendicular to those of the neighboring links), the last four links had a unique rotation axis. To validate the proposed control system using the $P^{5}R$ snake-like robot, each of the constraint models developed in Section 3 was implemented as part of a motion and trajectory control software, as shown in Figure 6. The control system had two basic modules, which were developed for motion and trajectory control of the flexible snake-like robot. The motion control module consisted of snippets that executed the kinematic and dynamic constraints control using the kinodynamic models in Section 3.2. Furthermore, the trajectory control module included the pre-coded procedures for executing the motion trajectory planning and obstacle avoidance models in Section 3.3. The software was built on the publicly available Robotics Toolbox [44] and it had a separate module for configuring different models of the flexible snake-like robots. The modules were designed with several buttons and input boxes for user interaction and conducting in silico simulation studies for the flexible robot.

Simulation of the robotic prototype and implementation of the control system, including the constraints models, were carried out using the Robotics Toolbox in the Matlab environment installed on a PC with an Intel^®^ Core i3 duo processor (2.40 GHz each) and 2 GB RAM. An in silico experiment was set up to validate the control system based on vital objectives, such as navigation accuracy, execution time, and path reachability, when each of the constraints’ models were sequentially executed for path navigation exercise. In the study, the simulated $P^{5}R$ snake-like robot was to navigate along an arbitrary circular path that consisted of *n* points that were equally spaced in an X-Y plane but tilted along a corresponding z-axis. Coordinates of each data point were parameterized using Equation (26):

(26) $${\left(x-19\right)}^{2}+{\left(y-2\right)}^{2}+{\left(z-13\right)}^{2}=10.$$

The control study was followed with sequential execution of the models for kinodynamic constraints and trajectory tracking in an attempt to ensure a collision-free point-to-point navigation. The number of data points were varied over three different trials, and coordinates of the data points were iteratively set as the target point in each trial.

Finally, it was observed that the proposed control system navigated the flexible snake-like robot around the three circular paths with highly accurate kinematics and a fast dynamics resolution during the point-to-point navigation, and the collision-free potential of the control system was also guaranteed. Detailed results obtained from each of the kinematics, dynamics, trajectory planning, and collision avoidance algorithms are presented below.

### 4.1. Results of the IK Model

**Figure 7:**
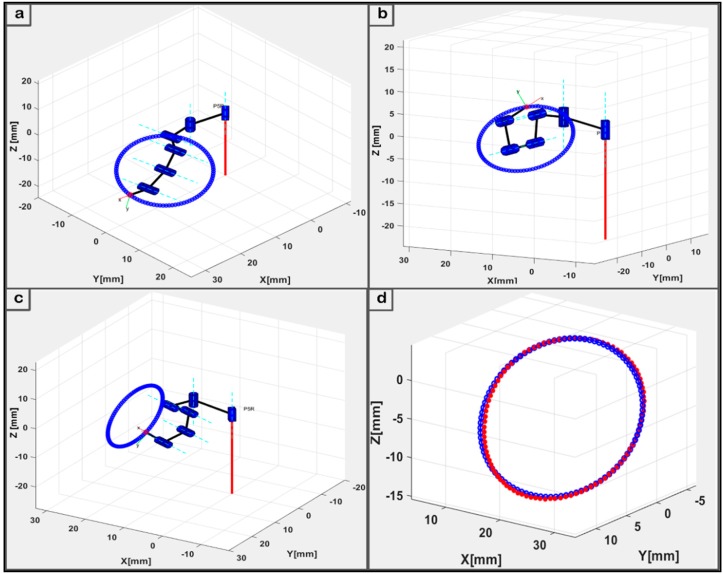
The IK results for the three case scenarios of a 3D circular path with *n* via-points: (**a**) description of *n* = 1; (**b**) description of *n* = 10; and (**c**) description of *n* = 20; and (**d**) Plot of actual and compute target points.

The operation of the flexible snake-like robot used to navigate a desired trajectory started with setting coordinates of each via-point in the circular path as an input of the proposed geometric IK model. This part of the program computed appropriate joint angles with which the robot tip could be positioned at each via-point in the path. Each navigation scenario had different number (*n*) of via-points, and the joint vectors obtained with the IK model were used to navigate the robot’s last link through the via-points. The poses obtained for the three scenarios (*n* = 1, 10, and 20) are shown in Figure 7a–c. A major objective was simultaneously solving the IK in a very accurate and fast manner for timely online motion control. For accuracy, limited axial offsets could be tolerated between the desired points along a path and the actual points the robot could reach when using the IK solution. The kinematic model was first validated with error offsets during each circular path tracking trial. The kinematic error values were obtained as the difference between the axial values of each data point and the corresponding actual point obtained using the IK model. We applied forward kinematics to determine the actual coordinates of the last link’s tip when the joint angles computed by our proposed method were set as the input.

A comparison between the desired via-points and the actual points obtained when the geometric IK technique was used during the circular path tracking is presented as Figure 7d. The blue and red points are the desired via-points generated using Equation (11) and the actual points computed from the IK model, respectively. By analyzing the kinematic results obtained when the 3D circular paths were tracked by the flexible robot, it was observed that the IK model had accurately reached more than 90% of all points in the circular path with 100 via-points; that is, *n* = 1. With a kinematics error threshold of 1 mm, an average kinematics error of 0.37 ± 0.36 mm was obtained from the 100 points with a 92% reachability. Besides the error offset analysis, the performance of the kinematic constraint model was also observed in terms of the model’s run-time taken to resolve the IK for each point. The run-time was the average execution time the model took to compute the IK of a single point over all the points in each circular movement. The geometric IK method had a maximum average response time of 0.061 s over all three trials. This was significantly close to zero relative to the time taken to reach each point in the six-link robot’s workspace. To the best of our knowledge, there exists no single method that has solved the IK of such a modular configuration with both a high accuracy and a short computational time simultaneously.

### 4.2. Result of the Dynamics Model

During the point-to-point navigation, the displacements of all joints in the robot were taken from the geometric IK model and set as the input of the recursive Newton–Euler model. The higher-order motion variables of the robots, namely velocities and accelerations, were computed and utilized in forward and backward recursions of the dynamics model to determine the generalized forces acting on the robot’s links. To ensure consistency between the simulated and real prototype of the robot, a compact and miniaturized design of the snake-like robot was parameterized using Solidworks^®^ 2016 (Dassault Systems Solidworks Corp., Waltham, MA, USA). This involved estimating the dynamic parameters of the cylindrical links with hollowed holes and pre-designed joints. The dynamic parameters, presented in Table 1, were obtained and employed for the implementation and validation of the recursive Newton–Euler model. The values were obtained with the links’ center of mass aligned with their corresponding output coordinate system in the Solidworks^®^ environment. Furthermore, a constant mass density was assumed for each link, and their geometric centers were equally taken to be exactly each link’s center of mass. The joints of the robot were actuated using a Faulhaber^®^ DC (Clearwater, FL, USA) micromotor with a rotor inertia of 5.7 g·mm^2^. With these, the simulated model of the robot’s links exhibited similar parameters as the proposed prototype. The computations started by deriving up to the second-order motion and force profiles using every consecutive joint position variable of the robot as it navigated the paths. For this purpose, the joint positions obtained during the execution of the kinematic model were differentiated to determine each joint’s velocity and acceleration while the corresponding forces at both terminal ends of the robot were obtained.

**Figure 8:**
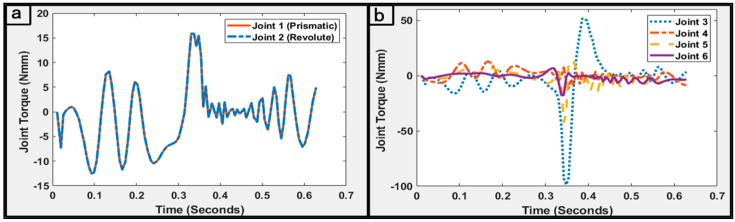
Dynamic analysis of the generalized forces in the robotic mechanism for the trajectory case where *n* = 10 (**a**)joint torque at the first two links; and (**b**) joint torque at the last four links.

The latter was done by propagating zero initial velocities and acceleration outward from the stationary base link of the robot, while the terminal force and moment at the last link were set to zero. Thus, with other required parameters required by the dynamic model, the generalized forces acting at each joint of the robot during the point-to-point circular motion were computed as shown in Figure 8. It can be observed that the consecutive transitions of the robot’s joints over all the consecutive via-points were relatively steady for the last four revolute joints. This could be attributed to the highly accurate IK constraints with the resolution achieved in a timely manner.

In addition, a mean execution time of 0.057 s was required for the iterative execution of the dynamic model. Thus, the model could enhance the real-time control of the robot. Similarly, analysis of the torque reflecting at the robot’s joints showed a high symmetry of the generalized forces amongst the four joints, except for the joint connecting the third link. This could be explained with respect to the role of the joint in enhancing the spatially flexible movement of the snake-like robot. The plot in Figure 8b shows that comparably smaller torque inputs were required by the first two joints and the last link. Both links in the former had approximately similar generalized forces and this was because the prismatic joint had a limited offset and hardly moved during the trajectory. However, for the latter, the smaller torque was associated with the desired value set for the joint, and thus, the effects of gravity are reduced when the link was used for surgical contact. The peculiar structure of the kinematic and dynamic model was keenly exploited for implementation of the trajectory planning and collision avoidance techniques.

### 4.3. Motion Trajectory Model Results

As presented above, results from the kinematics and dynamics model were plausible for the navigation along the given paths. The points in each path were assigned as an admissible motion trajectory as computed by the planning strategy developed in Section 3.3. The selection of the trajectory control basis was based on the flexibility of the robot’s structure and navigation along the circular path. Thus, sequences of the via-points were referenced in joint space for the trajectory planning, while the $P^{5}R$ robot tracked the circular path in a point-to-point manner. The three scenarios of *n* via-points (i.e., *n* = 1, 10, and 20) were studied to validate the trajectory constraints model across the time splines. For convenience, the sequence of points obtained for each case was assigned and the corresponding joint variables computed in the workspace were followed for proper monitoring of the executed trajectories during navigation. The via-points were validated using the motion profile obtained when the cycloidal trajectory model in Equations (21)–(23) was implemented.

**Figure 9:**
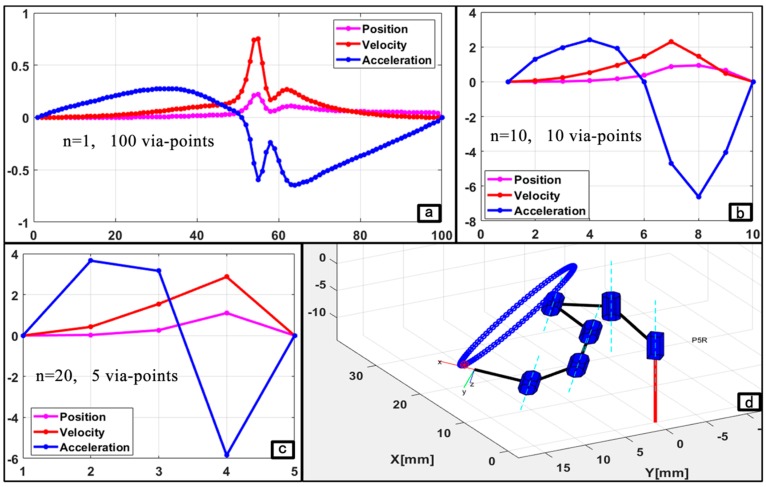
Motion profiles of the robot: (**a**–**c**) trajectory results for the three cases and (**d**) the trajectory error.

Table 2 shows the results obtained for the trajectory planning when executed for the case of *n* = 20 via-points. This includes the coordinates of the last link, as well as the positions, velocities, and accelerations of all joints and their time splines during the point-wise movement. For each consecutive $P_{i}$ → $P_{j}$ movement in the three trajectory cases, the pose of the saddle points comprised a unit vector with orientation $R_{i,j}$ = ${\left[R_{x},\;R_{y},\;R_{z}\right]}^{T}$ defined along the normal of each via-point ($P_{i}$) in the flexible robot’s workspace. Plots in Figure 6 show the poses of the robot along with an instance of the joint displacement during its navigations in the three scenarios. The motion was primed with values of variables ${\dot{q}}_{j,1}$, ${\dot{q}}_{j,n}$, ${\ddot{q}}_{j,1}$, and ${\ddot{q}}_{j,n}$ set to be zero for all joints *j* = 1, 2,…, 6. The robotic model was initialized to move along the circular path from rest and came to a full stop at the end of the trajectory. Joint displacements from the initial point through the desired via-points to the target point were computed in joint space by solving the robot’s IK. The model described for a normalized profile of the motion’s trajectory was implemented for the three cases of the circular path. For convenience, the sequence of points in each case was assigned and the corresponding joint variables computed in the workspace were followed for proper monitoring of the executed trajectories during navigation. Motion profiles for each of the three motion cases are presented in plots of Figure 9a–c.

Figure 9 shows that the control system demonstrated geometrically and parametrically continuous motion with smooth trajectories in all the cases. Furthermore, the motion duration remained stable at approximately 0.623 s, and the workspace boundary and interior singularities were not encountered while navigating the circular pathways. Since trajectory error is an important metric for developing a motion control system for flexible robotic surgery, we further analyzed the point-to-point navigations to observe the error displacement for a given point in the trajectory. For instance, Figure 7d shows the error details in the circular trajectory tracking with 100 via-points.

### 4.4. Results from the Obstacle Avoidance Model

**Figure 10:**
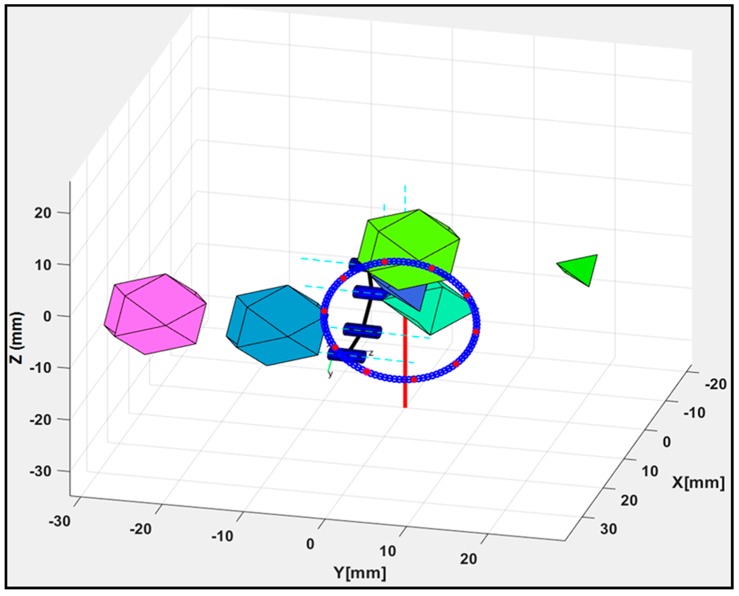
Obstacle avoidance of the robot for the trajectory case with *n* = 10.

Objects that are not of interest in a workspace or links making-up the flexible robot can be deemed as obstacles, and these can hinder the steady or continuous motion of a robotic mechanism when moving along a pathway with a pre-planned trajectory, which reduces the efficiency of the trajectory planning system. The avoidance of collision with obstacles in the workspace was initiated during the point-to-point navigation trials. For this purpose, polyhedral-shaped objects were randomly introduced as workspace obstacles while the trajectory model was executed. The interpolation of the robot’s links with each obstacle in the workspace was checked and the PoC for the potential collision points were calculated in accordance with Section 3.3. Since each polygonal area was encircled, the algorithm checked for interpolation using the center and radius of both circles to decide whether a collision with an obstacle may occur and to trigger any necessary avoidance. Again, Equation (10) was used to generate a circular path while tracking scenarios with multiple obstacles were established to validate the avoidance model. Figure 10 shows a scenario with five different obstacles in the robot’s workspace. Collision-free movement was obtained by using poses with the value of *PoC* ≈ 0 from the pool of the robot’s poses generated using the IK scheme in Section 3.2 as the robot moved along with the desired trajectory in the circular paths.

The detection algorithm of the model compiled PoC values for all link–obstacle pairs in the robot’s workspace, and the h value in Figure 3a was changed to determine an IK solution that avoided collisions with obstacles during each point-to-point movement. Thus, the circular path was tracked and none of the trajectory movements were ensnared by an obstacle during the point-to-point navigation trials. The proposed system found optimized joint angles for all links to stably track the trajectory. Furthermore, components of the motion control system were evaluated to analyze the performance of the collision avoidance strategy. For this purpose, execution times when the control system was used with and without the collision avoidance strategy were compared and average execution times of 0.019 ± 0.008 s and 3.576 ± 1.688 s were obtained, respectively. Thus, the strategy used had a relatively high computational complexity; in fact, the time complexity directly depended on the number of interpolated points found when executing the obstacle detection and avoidance algorithm. Furthermore, the algorithm optimally maintained a safe distance between the robot’s links and the obstacles in the workspace.

## 5. Conclusions and Future Works

Recent developments in robotic surgery include designing flexible mechanisms that can enhance surgical interventions, such as suturing, tumor resection, and even radio-surgical interventions. In this study, a control system was proposed for the motion and trajectory tracking of flexible robots in this paper. This included a $P^{n}R$ snake-like robotic mechanism designed for the navigation of a flexible surgical pathway, and the derivation of kinematic and dynamic constraint models for safe motion and trajectory control of the snake-like mechanism. Furthermore, the proposed surgical platform and control system were validated in silico. According to the implementation and validation results, the snake-like model was capable of navigating a flexible pathway, and the proposed motion and trajectory control system could quickly and accurately navigate the surgical robot. The IK resolution model in the control system was based on geometric analysis of the robot’s links and joints in Cartesian space, while the dynamic constraints modeling was focused on computing the generalized force acting at the robot’s links and joints during the path navigation. Both models demonstrated viably fast resolutions—6.1 ms for kinematics and 5.7 ms for dynamics—when the robotic mechanism was used to navigate three instances of a circular pathway. Kinematically, the proposed model demonstrated a 92% reachability when an error threshold of 1 mm was used. Thus, enhancement of the optimal trajectory tracking was achieved with the flexible snake-like robot in the circular-path-tracking study. The fast and efficient control system benefited from using simple Cartesian analytics for the model derivation. Hence, the control system can be ideally used for surgical robotic teleoperation.

In the future, another experimental validation will be performed using the same proposed robotic design. For this purpose, the proposed constraints control system will be enhanced to fit the complexity of the flexible snake-like robot such that each revolute joint will have a unique rotation axis, as in Figure 1. Furthermore, the dynamics model, which is currently based on only the first two derivatives of the position data, will be improved and high-order generalized forces acting at the robot’s joints will also be considered. To avoid the noisy moment and torque values generated using the recursive Newton–Euler model, appropriate signal filtering model will be incorporated in a future study. Lastly, intelligence-based control methods will also be adapted for the kinematics and dynamics resolution, as well as the obstacle collision detection and avoidance schemes.

## Figures and Tables

**Table 1:** Dynamic parameters of the snake-like robot.

Link ID	Mass (g)	Center of Mass (mm)			Moment of Inertia (g·cm^2^)					
		X	Y	Z	$l_{xx}$	$l_{yy}$	$l_{zz}$	$l_{xy}$	$l_{xz}$	$l_{yz}$
1	16.44	0.07	0.45	−5.98	181.24	181.30	9.58	0.39	4.55	1.20
2	20.07	0.26	0.24	−14.73	64.87	65.04	11.41	−0.04	2.89	−2.59
3	7.12	0.11	0.45	−15.80	16.90	16.91	4.39	0.2	0.43	0.80
4	7.12	0.12	0.45	−22.70	16.88	16.90	4.40	0.32	0.81	0.53
5	7.12	0.14	0.52	−29.11	16.89	16.90	4.38	0.31	0.64	0.42
6	7.93	0.40	0.59	−35.97	23.12	23.09	4.91	0.08	−0.62	1.43

**Table 2:** Parametric description of the robot for motion case 3.

Id	Time (ms)	Last Link Frame (mm)			Joint Displacements (rad)						Velocity (mm/s)	Acc (mm^2^/s)
		X	Y	Z	${ɗ}^{*}$	φ	$\theta_{1}$	$\theta_{2}$	$\theta_{3}$	$\theta_{4}$		
1	0.005	29.95	2.95	−10.39	0.0	0.14	1.79	−0.79	−0.55	−0.79	0.00	0.00
2	0.011	22.39	11.97	−11.84	−5.3^−17^	0.17	1.81	−0.79	−0.55	−0.79	0.42	3.65
3	0.015	14.37	11.28	−3.14	−1.1^−15^	0.88	2.22	−0.91	−0.79	−0.91	1.55	3.16
4	0.023	17.18	1.85	3.45	−8.7^−17^	0.16	0.95	−1.19	−1.84	−1.19	2.86	−5.85
5	0.029	26.86	−3.05	−1.29	0.0	0.25	1.09	−1.18	−1.79	−1.18	0.00	0.00

* Final offset value obtained during the IK resolution for a given target point.

## References

[1] Beasley R. (2012). Medical Robots: Current Systems and Research Directions. J. Robot..

[2] Omisore O., Han S., Ren L., Wang G., Ou F., Li H., Wang L. (2018). Towards Characterization and Adaptive Compensation of Backlash in a Novel Robotic Catheter System for Cardiovascular Intervention. IEEE Trans. Biomed. Circuits Syst..

[3] Omisore O., Han S., Ren L., Elazab A., Azeez N., Talaat T., Wang L. (2018). Deeply-Learnt Damped Least-Squares Method for Inverse Kinematics of Snake-like Robots. Neural Netw..

[4] Schmitz A., Berthet-Rayne P., Yang G.-Z. Endoscopic Bi-Manual Robotic Instrument Design Using a Genetic Algorithm. Proceedings of the IEEE International Conference on Intelligent Robots and Systems.

[5] Simaan N., Yasin R.M., Wang L. (2018). Medical Technologies and Challenges of Robot-Assisted Minimally Invasive Intervention and Diagnostics. Annu. Rev. Control. Auton. Syst..

[6] Seah T., Do T., Takeshita N., Ho K., Phee S. (2017). Future of flexible robotic endoscopy systems, Diagnostic and Therapeutic Procedures in Gastroenterology. arXiv.

[7] Omisore O., Han S., Ren L., Zhang N., Ivanov K., Elazab A., Wang L. (2017). Non-iterative geometric approach for inverse kinematics of redundant lead-module in a radiosurgical snake-like robot. BioMed. Eng. OnLine.

[8] Su H., Yang C., Mdeihly H., Rizzo A., Ferrigno G., de-Momi E. (2019). Neural Network Enhanced Robot Tool Identification and Calibration for Bilateral Teleoperation. IEEE Access.

[9] Su H., Yang C., Ferrigno G., de Momi E. (2019). Improved Human–Robot Collaborative Contro of Redundant Robot for Teleoperated Minimally Invasive Surgery. IEEE Robot. Autom. Lett..

[10] Wang W., Li Y. Path Planning for Redundant Manipulator Without Explicit Inverse Kinematics Solution. Proceedings of the IEEE International Conference on Robotics and Biomimetics.

[11] Yahya S., Moghavvemi M., Mohamed H. (2011). Geometrical Approach of Planar Hyper-Redundant Manipulators: Inverse Kinematics, Path Planning and Workspace. Simul. Model. Pract. Theory.

[12] Agarwal V. (2012). Trajectory Planning of Redundant Manipulator using fuzzy Clustering Method. Int. J. Adv. Manuf. Technol..

[13] Omisore O., Han S., Ren L., Zhang N., Wang L. A Geometric Solution for Inverse Kinematics of Redundant Teleoperated Surgical Snake Robots. Proceedings of the IEEE International Conference on Industrial Technology.

[14] Luh J., Walker M., Paul R. (1980). On-line computational scheme for mechanical manipulators. J. Dyn. Syst. Meas. Control.

[15] Saha S.K. (1999). Dynamics of serial multibody systems using the decoupled natural orthogonal complement matrices. J. Appl. Mech..

[16] Hurst J., Chestnutt J., Rizzi A. (2010). The actuator with mechanically adjustable series compliance. IEEE Trans. Robot..

[17] Vera F. (2016). Modeling and Sliding-Mode Control of Flexible-Link Robotic Structures for Vibration Suppression.

[18] Rodriguez-Cianca D., Weckx M., Jimenez-Fabian R., Torricelli D., Gonzalez-Vargas J., Sanchez-Villamañan M., Sartori M., Berns K., VanderBorght B., Pons J.L. (2019). A Variable Stiffness Actuator Module With Favorable Mass Distribution for a Bio-inspired Biped Robot. Front. Neurorobotics.

[19] Simaan N., Taylor R., Flint P. High Dexterity Snake-Like Robotic Slaves for Minimally Invasive Telesurgery of the Upper Airway. Proceedings of the International Conference on Medical Image Computing and Computer-Assisted Intervention.

[20] Franzino R.J. (2003). The Laprotek surgical system and the next generation of robotics. Surg. Clin..

[21] Abbott D.J., Becke C., Rothstein R.I., Peine W.J. Design of an Endoluminal NOTES Robotic System. Proceedings of the IEEE/RSJ International Conference on Intelligent Robots and Systems.

[22] Degani A., Choset H., Wolf A., Ota T., Zenati M.A. Percutaneous Intrapericardial Interventions Using a Highly Articulated Robotic Probe. Proceedings of the 1st IEEE International Conference on Biomedical Robotics and Biomechatronics.

[23] Ota T., Degani A., Schwartzman D., Zubiate B., McGarvey J., Choset H., Zenati M.A. (2009). A highly articulated robotic surgical system for minimally invasive surgery. Ann. Thorac. Surg..

[24] Thakkar S., Awad M., Gurram K.C., Tully S., Wright C., Sanan S., Choset H. (2015). A Novel, New Robotic Platform for Natural Orifice Distal Pancreatectomy. Surg. Innov..

[25] Carmichael J. Novel Robotic Techniques for Endoscopic Resection of Large Polyps. Proceedings of the 10th Annual Gastroenterology and Hepatology Symposium.

[26] Garriga-Casanovas A., Baena F. (2018). Complete follow-the-leader kinematics using concentric tube robots. Int. J. Robot. Res..

[27] Jain A. (1991). Unified Formulation of Dynamics for Serial Rigid Multibody Systems. J. Guid. Control. Dyn..

[28] Ali S. (2011). Newton-Euler Approach for Bio-robotics Locomotion Dynamics: From Discrete to Continuous Systems. Ph.D. Thesis.

[29] Dong K., Liu H., Zhu X., Wang X., Xu F., Liang B. (2019). Force-free control for the flexible-joint robot in human-robot interaction. Comput. Electr. Eng..

[30] Omisore O., Han S., Ren L., Zhao Z., Al-Handarish Y., Igbe T., Wang L. A Teleoperated Snake-Like Robot for Minimally Invasive Radiosurgery of Gastrointestinal Tumors. Proceedings of the 18th IEEE ICARSC.

[31] Lozano-Perez T. (1983). Spatial Planning: A Configuration Space Approach. IEEE Trans. Comput..

[32] Angeles J., Rojas A., Lopez-Cajun C. (1998). Trajectory Planning in Robotic Continuous Path Application. IEEE J. Robot. Autom..

[33] Parsa S., Daniali H., Ghaderi R. (2010). Optimization of Parallel Manipulator Trajectory for Obstacles and Singularity Avoidances Based on Neural Network. Int. J. Adv. Manuf. Technol..

[34] Dasgupta B., Gupta A., Singla E. (2009). A Variational Approach to Path Planning for Hyper-Redundant Manipulators. Robot. Auton. Syst..

[35] Kuntz A., Mahoney A.W., Peckman N.E., Anderson L., Maldonado F., Webster R.J., Alterovitz R. Motion Planning for Continuum Reconfigurable Incisionless Surgical Parallel Robots. Proceedings of the IEEE/RSJ International Conference on Intelligent Robots and Systems.

[36] Liu Q., Wang C.-B., Zhang B., Duan L., Zhang X., Sun T., Shang W., Shen Y., Lin Z., Li X. Kinematics analysis and motion planning of a redundant robotic manipulator for surgical intervention. Proceedings of the 2017 IEEE International Conference on Cyborg and Bionic Systems (CBS).

[37] Sun W., Torres L.G., van den Berg J., Alterovitz R. (2016). Safe Motion Planning for Imprecise Robotic Manipulators by Minimizing Probability of Collision. Robotics Research.

[38] Su H., Ovur S., Zhou X., Qi W., Ferrigno G., de Momi E. (2020). Depth vision guided hand gesture recognition using electromyographic signals. Adv. Robot..

[39] Du W., Omisore O., Duan W., Zhou T., Lv X., Li Y., Han S., Al-Handarish Y., Liu Q., Wang L. (2020). Exploration of Interventionists’ Technical Manipulation Skills for Robot-Assisted Intravascular PCI Catheterization. IEEE Access.

[40] Su H., Qi W., Yang C., Sandoval J., Ferrigno G., de-Momi E. (2020). Deep Neural Network Approach in Robot Tool Dynamics Identification for Bilateral Teleoperation. IEEE Robot. Autom. Lett..

[41] Chen Y., Xu W., Li Z., Song S., Lim C., Wang Y., Ren H. (2017). Safety-Enhanced Motion Planning for Flexible Surgical Manipulator Using Neural Dynamics. IEEE Trans. Control Syst. Technol..

[42] Berni A., Ramdane-Cherif A., Saadia N., Levy N. Exploring Cognitive Approach Through the Neural Network Paradigm: Trajectory Planning Application. Proceedings of the IEEE International Conference on Cognitive Informatics.

[43] Mayorga R., Aduthaya T. An ANN Approach for the Motion Planning of Redundant Manipulators. Proceedings of the International Conference on Systems, Control & Informatics.

[44] Chen Z., Su W., Li B., Deng B., Wu H., Liu B. (2018). An intermediate point obstacle avoidance algorithm for serial robot. Adv. Mech. Eng..

[45] Xu Z., Zhou X., Li S. (2019). Deep Recurrent Neural Networks Based Obstacle Avoidance Control for Redundant Manipulators. Front. Neurorobotics.

[46] Park B., Oh H. (2020). Vision-Based Obstacle Avoidance for UAVs via Imitation Learning with Sequential Neural Networks. Int. J. Aeronaut. Space Sci..

[47] He W., Yan Z., Sun Y., Ou Y., Sun C. (2018). Neural-Learning-Based Control for a Constrained Robotic Manipulator With Flexible Joints. IEEE Trans. Neural Netw. Learn. Syst..

[48] Fahimi F., Ashrafiuon H., Nataraj C. (2003). Obstacle Avoidance for Spatial Hyper-Redundant Manipulators using Harmonic Potential Functions and the Mode Shape Technique. J. Robot. Syst..

[49] Omisore O., Han S., Al-Handarish Y., Du W., Duan W., Wang L. Motion and Trajectory Control System for Flexible Robots in Minimally Invasive Surgery. Proceedings of the 4th International Conference on Mechanical Engineering and Robotics Research.

[50] Hong I. (2015). Deriving an Obstacle-Avoiding Shortest Path in Continuous Space: A Spatial Analytic Approach. Ph.D. Thesis.

[51] Corke P. (2017). Robotics, Vision and Control: Fundamental Algorithms. MATLAB.

